# Understanding Ocular Surface Disease in Glaucoma: A Comparative Analysis of Symptoms and Objective Parameters

**DOI:** 10.7759/cureus.54070

**Published:** 2024-02-12

**Authors:** João Romano, Nélia Ferreira, Gonçalo Godinho, Rita Tomás, Nuno Oliveira, João Paulo Sousa

**Affiliations:** 1 Ophthalmology, Centro Hospitalar de Leiria, Leiria, PRT

**Keywords:** topical intraocular pressure-lowering therapies, primary open-angle glaucoma, tear film break-up time, non-invasive tear break-up time, glaucoma, ocular surface disease, osdi questionnaire, keratograph

## Abstract

Background

Glaucoma is a progressive optic neuropathy that may result in irreversible visual impairment and can diminish quality of life. Lowering intraocular pressure (IOP) through topical eyedrops is usually the primary approach to managing glaucoma. However, long-term treatment poses a risk to ocular surface health, leading to ocular surface disease (OSD). Preservative-containing eyedrops are implicated in OSD development due to their detrimental effects on the tear film and goblet cell density. OSD symptoms may impact patient compliance due to local side effects. This study aims to assess OSD in glaucoma patients receiving topical treatment, quantify symptoms and objective ocular surface parameters, and compare them to a control group not using topical glaucoma medications.

Methodology

Patients diagnosed with primary open-angle glaucoma receiving topical treatment and a control group were included in this study. To assess OSD, patients completed the Ocular Surface Disease Index (OSDI) questionnaire to evaluate symptoms and underwent objective measurements of ocular surface parameters using a keratograph. These parameters included assessments of bulbar redness and non-invasive keratograph tear break-up time (NIKTBUT).

Results

A cohort of 92 patients was subjected to examination, comprising 66 individuals diagnosed with glaucoma and 26 controls. Within the glaucoma patient subset, the mean number of IOP-lowering drugs administered was 2.42 ± 0.18, with 22.7% exclusively utilizing preservative-free eye drops. Our investigations unveiled a substantial prevalence of OSD symptoms, manifesting not only within the glaucoma cohort but also among the control group, with 72.7% and 53.8%, respectively (p = 0.224), reporting moderate-to-severe symptoms (OSDI > 23). Remarkably, OSDI scores exhibited higher values among female participants (p = 0.039) and glaucoma patients using prostaglandins (p<0.001) and were negatively correlated to the number of IOP-lowering drugs used (-0.448; p < 0.001). Furthermore, employing keratograph assessment, we discerned heightened bulbar redness (1.86 ± 0.07) in the glaucoma group compared to the control group (1.58 ± 0.07; p = 0.008). Glaucoma subgroup analyses further unveiled higher bulbar redness among glaucoma patients employing carbonic anhydrase inhibitors (p = 0.035) and applying medication preservatives (p = 0.045) but lower among individuals using beta‐blockers (p = 0.018). However, the NIKTBUT did not show significant variance between the two groups (glaucoma group: 10.19 ± 0.85 seconds; control group: 10.96 ± 1.37 seconds; p = 0.499).

Conclusions

Our study revealed a significant prevalence of OSD in our sample, with the OSDI questionnaire showing limited specificity. The notable increase in bulbar redness pointed to an elevated prevalence of OSD among glaucoma patients, emphasizing the considerable impact of preservatives on ocular surface damage. Recognizing the potential damage to the tear film and ocular surface is crucial for glaucoma experts, who must employ comprehensive therapeutic strategies to mitigate symptoms, advocating for the preferential use of preservative-free medications, when possible, for optimizing long-term treatment.

## Introduction

Glaucoma is a progressive optic neuropathy that poses a significant risk of irreversible visual impairment and adversely impacts the quality of life [1]. Management primarily involves the lifelong use of topical eyedrops to lower intraocular pressure (IOP). Ocular surface disease (OSD), a prevalent reason for ophthalmologist visits and commonly associated with glaucoma, manifests as inadequate tear quantity, an unstable tear film, and various discomforting symptoms [2]. These symptoms, including irritation, burning, dryness, and fluctuating visual acuity, can profoundly affect patients’ well-being and work capabilities [2].

The long-term use of topical glaucoma eyedrops, particularly those containing preservatives, poses a potential threat to ocular surface health and potentially impacts patient compliance due to local side effects [3,4]. They are implicated in OSD development by affecting the tear film composition and meibomian gland function, reducing goblet cell density, and increasing proinflammatory cytokines (IL-6, IL-8, and IL-10) [5,6].

The widely used Ocular Surface Disease Index (OSDI) questionnaire addresses diverse aspects of OSD, including discomfort and functional limitations [7]. However, its multidimensional nature necessitates careful consideration when evaluating patients with concurrent ocular conditions such as glaucoma or cataracts [8]. Reduced visual acuity or visual field loss associated with glaucoma contributes to a lower self-reported quality of life, affecting specific tasks outlined in the OSDI questionnaire [1,8].

Objective analysis through keratography enables the assessment of ocular surface signs and precise quantification of tear film with proper documentation, yet studies in this field remain limited [9,10].

This study aims to comprehensively assess OSD in glaucoma patients undergoing topical treatment. Additionally, it seeks to quantify both symptoms and objective ocular surface parameters, comparing them with a control group not utilizing hypotensive therapy.

## Materials and methods

Patient selection

Patients attending the ophthalmic glaucoma subspecialty consultation at Centro Hospital de Leiria in Leiria, Portugal, were invited to participate in this study (glaucoma group). Inclusion criteria encompassed patients diagnosed with primary open-angle glaucoma who had been using at least one topical hypotensive drug for at least six months with no previous diagnosis of OSD. The control group included patients attending the preoperative cataract surgery consultation at Centro Hospital de Leiria who were not undergoing glaucoma treatment with no previous diagnosis of OSD. Patient confidentiality was strictly maintained throughout the study. This research adhered to the ethical principles outlined in the Declaration of Helsinki. Approval for the study was obtained from the local ethics committee (Centro de Investigação da Unidade de Local de Saúde da Região de Leiria, approval number: Ata nº10 de 2023.12.14).

Ocular Surface Disease Index

The OSDI questionnaire was administered to all participants by a masked ophthalmic technician. The 12-item questionnaire was translated and validated in Portuguese by Prigol et al. [11]. Individual OSDI questions were scored on a four-point Likert scale, with scores ranging from 0 to 4 (none of the time, some of the time, half of the time, most of the time, and all of the time, respectively). The total OSDI score was calculated (OSDI = [(sum of the score for all the questions answered)×100]/[(12- total number of questions not answered)×4]), as well as a sub-analysis, including OSDI vision-related score (only considering 4-9 items) and OSDI ocular discomfort-related score (only considering 1-3 and 10-12 items) obtained using the same formula [8]. Total scores ranged from 0 to 100 (normal: 0-12; mild: 13-22; moderate: 23-32; severe: 33-100) and sub-analysis scores ranged from 0 to 50.

Keratograph analysis

Keratograph 5M (Oculus, Wetzlar, Germany) was used to perform non-invasive and objective ocular surface analysis without requiring anesthesia or stains. For each eye, using infrared light video, the keratograph software generated measurements (time in seconds) of the first non-invasive keratograph tear break-up time (NIKBUT first) as well as its average time (NIKBUT avg). Bulbar redness was also automatically assessed and scored using digital imaging of the anterior biomicroscopy.

Statistical analysis

The variables were described using mean and standard deviation (SD). The normality of quantitative variables was evaluated using the Kolmogorov-Smirnov and Shapiro-Wilk tests. When these assumptions were met, independent samples were analyzed using the t-test. If these assumptions were not satisfied, the Mann-Whitney test was applied. A p-value less than 0.05 was considered statistically significant.

## Results

A cohort of 92 patients was subjected to examination, comprising 66 individuals diagnosed with glaucoma and 26 controls. The mean age of the participants was 72.54 ± 1.33 years and 55.4% were males (Table 1). There was no significant difference between age and sex between both groups (p = 0.922 and p = 0.114, respectively). Within the glaucoma patient subset, the mean number of IOP-lowering drugs administered was 2.42 ± 0.18, with 22.7% exclusively utilizing preservative-free eye drops.

**Table 1:** Patient demographics in the glaucoma and control groups. *: n = 132; **: n = 52. The Mann-Whitney test was employed to determine statistical significance. Bold values indicate statistically significant findings at p < 0.05. SD = standard deviation

	Glaucoma group (n = 66)	Control group (n = 26)	P-value
Age (years, mean ± SD)	72.70 ± 1.54	72.15 ± 2.70	0.922
Sex (male, n)	40 (60.6%)	11 (42.3%)	0.114
Race (Caucasian, n)	66 (100%)	26 (100%)	1
Phakic eyes (n)	52 (39.4%)*	44 (84.6%)**	<0.001

Our investigations unveiled a substantial prevalence of OSD symptoms, manifesting not only within the glaucoma cohort but also among the control group, with 72.7% and 53.8%, respectively (p = 0.224), reporting moderate-to-severe symptoms (OSDI > 23). The mean OSDI score (Table 2) did not reveal a statistically significant difference between glaucoma patients (34.13 ± 2.65) and the control group (31.65 ± 4.27, p = 0.677). The sub-analysis of the OSDI score also showed a non-significant difference between glaucoma patients and controls, including the vision-related OSDI (18.88 ± 1.64 and 17.92 ± 2.41, respectively; p = 0.760) and ocular discomfort-related OSDI (15.25 ± 1.57 and 13.72 ± 2.35, respectively; p = 0.598). Remarkably, total and discomfort-related OSDI scores exhibited higher values among female participants (p = 0.039 and p = 0.033, respectively). In glaucoma patients, it was also higher in those using prostaglandins (p < 0.001) and was negatively correlated to the number of IOP-lowering drugs used (-0.448; p < 0.001).

**Table 2:** Outcomes of Ocular Surface Disease Index scores and keratograph analysis in the glaucoma and control groups. Values are represented as mean ± standard deviation. The Mann-Whitney test was employed to determine statistical significance. Bold values indicate statistically significant findings at p < 0.05. Glaucoma group = total OSDI (0-77.08); vision-related OSDI (0-43.75); ocular discomfort-related OSDI (0-45.83); first NIKBUT (1.34-24.92); average NIKBUT (1.91-24.92). Control group = total OSDI (0-64.58); vision-related OSDI (0-41.67); ocular discomfort-related OSDI (0-33.33); first NIKBUT (3.25-24.92); average NIKBUT (4.62-24.92). OSDI = Ocular Surface Disease Index; NIKBUT = non-invasive keratograph tear break-up time

	Glaucoma Group (n = 66)	Control Group (n = 26)	P-value
Total OSDI (score)	34.13 ± 2.65	31.65 ± 4.27	0.677
Vision-related OSDI (score)	18.88 ± 1.64	17.92 ± 2.41	0.760
Ocular discomfort-related OSDI (score)	15.25 ± 1.57	13.72 ± 2.35	0.598
Bulbar redness (units)	1.86 ± 0.07	1.58 ± 0.07	0.008
First NIKTBUT (seconds)	10.19 ± 0.85	10.96 ± 1.37	0.499
Average NIKBUT (seconds)	15.27 ± 0.71	16.24 ± 1.02	0.474

Furthermore, employing keratograph assessment, we discerned heightened bulbar redness in the glaucoma cohort (1.86 ± 0.07) compared to the control group (1.58 ± 0.07; p = 0.008). Glaucoma subgroup analyses further unveiled higher bulbar redness among glaucoma patients using carbonic anhydrase inhibitors (p = 0.035) or applying medication preservatives (p = 0.045) and lower in individuals using beta‐blockers (p = 0.018).

Conversely, the first NIKTBUT did not exhibit a significant variance between the two groups (glaucoma group = 10.19 ± 0.85; control group = 10.96 ± 1.37; p = 0.499), nor did the average NIKTBUT (glaucoma group = 15.27 ± 0.71; control group = 16.24 ± 1.02; p = 0.474). Additionally, in the glaucoma subgroup analysis, there was no difference in the first and average NIKTBUT between patients using preservative and preservative-free glaucoma drops (p = 0.265 and p = 0.296, respectively).

## Discussion

The prevalence of OSD signs and symptoms was notably high among both the control and glaucoma groups in our study, surpassing findings from previous research [8-10,12]. Despite slight differences between the two groups, these distinctions did not reach statistical significance. This can be attributed to the potential inadequacy of the OSDI questionnaire in specifically discerning surface disease from other vision-related issues, such as cataracts, as it sums up all responses [8]. Even upon subscore analysis, the OSDI questionnaire proved insufficiently specific in distinguishing between both groups. Within the glaucoma cohort, we identified a higher score among patients using prostaglandins, contrary to the findings of some prior studies [13,14]. This might be attributed to our study’s lack of differentiation between monotherapy and combination therapy with other drugs. Notably, OSDI exhibited a negative correlation with the number of IOP-lowering drugs, aligning with previous research [8]. However, this association may be influenced not only by the cumulative effect of medications but also by the visual field defects in more advanced glaucomas requiring an intensified therapeutic intervention [8].

Our study noted a significant increase in bulbar redness, indicative of conjunctival inflammation potentially induced by OSD, in glaucoma-treated patients. Although various antiglaucoma medications are known to cause conjunctival hyperemia, our study highlighted the importance of preservatives as a significant contributor to ocular surface damage [9,10,12,15]. However, the lack of separate analysis of medications makes it challenging to compare specific drug effects with findings from other studies.

In contrast to some studies, we did not observe a difference in NIKTBUT between both groups [9,10]. Non-invasive objective methods prevent tear film disruption, eliminate reflex tearing, and facilitate comparisons between studies [9,10,16]. However, these measures are conducted under artificial conditions, with patients refraining from blinking, presenting challenges in patient cooperation and resulting in low repeatability [16].

Several limitations are present in our study, including a low number of participants, particularly in the control group. Additionally, a slightly higher proportion of females in the control group, who generally reported higher OSDI, may have introduced bias into the symptomatic analysis. The lack of separate analysis of IOP-lowering drugs prevents definitive conclusions in that regard. Furthermore, our study did not collect information on the duration of glaucoma therapy and the status of glaucoma, posing additional limitations. Future research with larger and more diverse cohorts, considering medication specifics and additional clinical parameters, is warranted to enhance our understanding of the intricate relationship between glaucoma treatment, OSD, and associated factors.

## Conclusions

In summary, our study uncovered a notable prevalence of OSD within our sample. The OSDI questionnaire exhibited limited specificity in distinguishing surface diseases from other vision-related issues. The significant increase in bulbar redness indicates a heightened prevalence of OSD among glaucoma patients, underscoring the significant contribution of preservatives to ocular surface damage. In these patients, the NIKTBUT did not demonstrate a significant decrease, but its acquisition presented challenges, underscoring the necessity for further validation and assessment of repeatability.

Considering that topical glaucoma medications may adversely impact ocular surface health, symptoms, and overall quality of life, glaucoma experts must be well-versed in recognizing potential signs of damage to the tear film and ocular surface. A comprehensive understanding of therapeutic strategies is crucial to restoring ocular surface homeostasis, enhancing tear film protective function, and alleviating the symptoms frequently reported by patients. Preferential use of preservative-free medications, when available, is advocated, particularly given the lifelong management requirements and the necessity for strict compliance in glaucoma treatment.
